# Genomic locus on chromosome 1 regulates susceptibility to spontaneous arthritis in mice deficiency of IL-1RA

**DOI:** 10.1186/s12865-014-0057-9

**Published:** 2014-12-09

**Authors:** Nan Deng, Yan Jiao, Yanhong Cao, Xiaoyun Liu, Yonghui Ma, Karen A Hasty, David D Brand, John M Stuart, Weikuan Gu

**Affiliations:** Department of Medicine, University of Tennessee Health Science Center, Memphis, TN 38163 USA; Departments of Orthopaedic Surgery and Biomedical Engineering, University of Tennessee Health Science Center, Memphis, TN 38163 USA; Mudanjiang Medical College, Mudanjiang, Heilongjiang 157001 PR China; Institute of Kaschin-Beck Disease, Center for Endemic Disease Control, Chinese Center for Disease Control and Prevention, Key Laboratory of Etiologic Epidemiology, Education Bureau of Heilongjiang Province & Ministry of Health (23618104), Harbin Medical University, Harbin, 150081 China; Research Service, Veterans Affairs Medical Center, 1030 Jefferson Avenue, Memphis, TN 38104 USA; Department of Microbiology, Immunology and Biochemistry, University of Tennessee Health Science Center, Memphis, TN 38163 USA

**Keywords:** Arthritis, Congenic breeding, Mouse, QTL, DBA/1

## Abstract

**Background:**

To understand the role of genetic factors on chromosome 1 in the regulation of spontaneous arthritis in mice deficient in IL-1 receptor antagonist protein (IL_1RA), we previously used speed congenic breeding to transfer the QTL region from DBA/1^−/−^ mice that are resistant to spontaneous arthritis into BALB/c^−/−^ mice which are susceptible. We were able to establish two congenic strains which exhibited a delayed onset and reduced severity of disease. In this study, we asked a different set of questions. How will the QTL region from BALB/c^−/−^ interact with the rest of the genome in the DBA/1^−/−^ background? Will the DBA/1^−/−^ mice become susceptible to spontaneous arthritis if the QTL genomic region on chromosome 1 was replaced with the genomic fragment of the same region from BALB/c^−/−^? We conducted the congenic breeding with the similar procedure as that of congenic strains with BALB/c^−/−^ background.

**Result:**

Instead of BALB/c^−/−^, DBA/1^−/−^ was used as the recurrent parent while BALB/c^−/−^ was used as the donor parent. By the 6^th^ generation we determined that all of the chromosomes in the progeny were of DBA/1^−/−^ origin with the exception of the QTL portion of chromosome 1 which is heterozygous of BALB/c^−/−^ and DBA/1^−/−^ origin. We then intercrossed selected mice to produce homozygous strains containing the homozygous genomic region of BALB/c^−/−^ on chromosome 1, while the rest of genome are homozygous DBA/1^−/−^. This strain was observed for the development of spontaneous arthritis. Up to 9 weeks of age, both congenic strain and DBA/1^−/−^ did not develop arthritis. However, after 9 weeks, the congenic strain started to exhibit signs of arthritis, while the DBA/1^−/−^ remained free from disease.

**Conclusion:**

The result indicates a strong influence of genetic factor(s) on the QTL of chromosome 1 on the susceptibility to spontaneous arthritis. Identification of genetic factors within this QTL region in the future will significantly enhance our understanding of molecular mechanism of spontaneous arthritis.

## Background

Elucidating the various processes involved in the development of inflammatory arthritis including RA has been greatly facilitated by the use of animal models. Interleukin-1 receptor antagonist (IL-1rn) -knockout BALB/c mice (BALB/c^−/−^) spontaneously develop autoimmunity and joint-specific inflammation that resembles human rheumatoid arthritis (RA) [1-3]. The development of arthritis inflammation is strain dependent. BALB/c mice that are homozygous for IL-1rn (BALB/c^−/−^) develop inflammation in the hind limbs with an incidence approaching 100% beginning at about 6 weeks of age. Histopathologic examination of the joints of these mice shows infiltration of inflammatory cells and synovial proliferation. However, DBA/1^−/−^ mice do not develop arthritis phenotype [3]. Detailed study of the molecular function of IL-1rn and its interaction with other genes or genetic factors is essential for development therapeutic application using IL-1rn. The genetic factors that interact with IL-1rn may be ideal targets for the development therapeutic applications.

In order to identify genetic factors that regulate spontaneous arthritis in BALB/c^−/−^, we used classical genetic techniques and bred susceptible and resistant mice to obtain an F2 generation and identified several QTL associated with arthritis susceptibility [4]. The QTL on chromosome 1 covers a large region at the distal end of the chromosome. We next conducted speed congenic breeding to transfer the QTL region from DBA/1^−/−^ mice that are resistant to spontaneous arthritis into BALB/c^−/−^ which are susceptible. We established two congenic strains with overlapping DBA/1^−/−^ DNA segments. These strains were observed for the development of spontaneous arthritis. Both congenic strains were relatively resistant to spontaneous arthritis and had delayed onset and a reduced severity of disease. The gene(s) that regulates this major QTL would appear to be located in the region of the QTL shared by both strains. The common transferred region is between D1Mit110 and D1Mit209 on chromosome 1 [5].

It is well known that disease phenotype is the final result of a complicated interaction between the genes in a genetic locus and environmental and genomic background in the congenic strain. The altered susceptibility to arthritis in the congenic strains under the BALB/c^−/−^genome background does not necessary mean that the locus will alter the disease susceptibility in a different background. Arthritis is a disease regulated by multiple genetic and environment factors. Genetic and genomic background of an individual plays an important role in the susceptibility to the disease. To investigate the effect of DBA^−/−^ genomic background on the function of genes in the QTL locus on chromosome 1 from BALB/c^−/−^, we conducted a congenic breeding using a similar procedure as with the congenic strains under the BALB/c^−/−^background. We transferred the genomic fragment from chromosome 1 of BALB/c^−/−^ into the DBA/1^−/−^ genomic background. We then used the congenic strain for the examination of the phenotype and potential candidate genes that regulate the susceptibility of the spontaneous arthritis.

## Results and discussion

### Congenic strain D1.BALB-1

**Figure 1:**
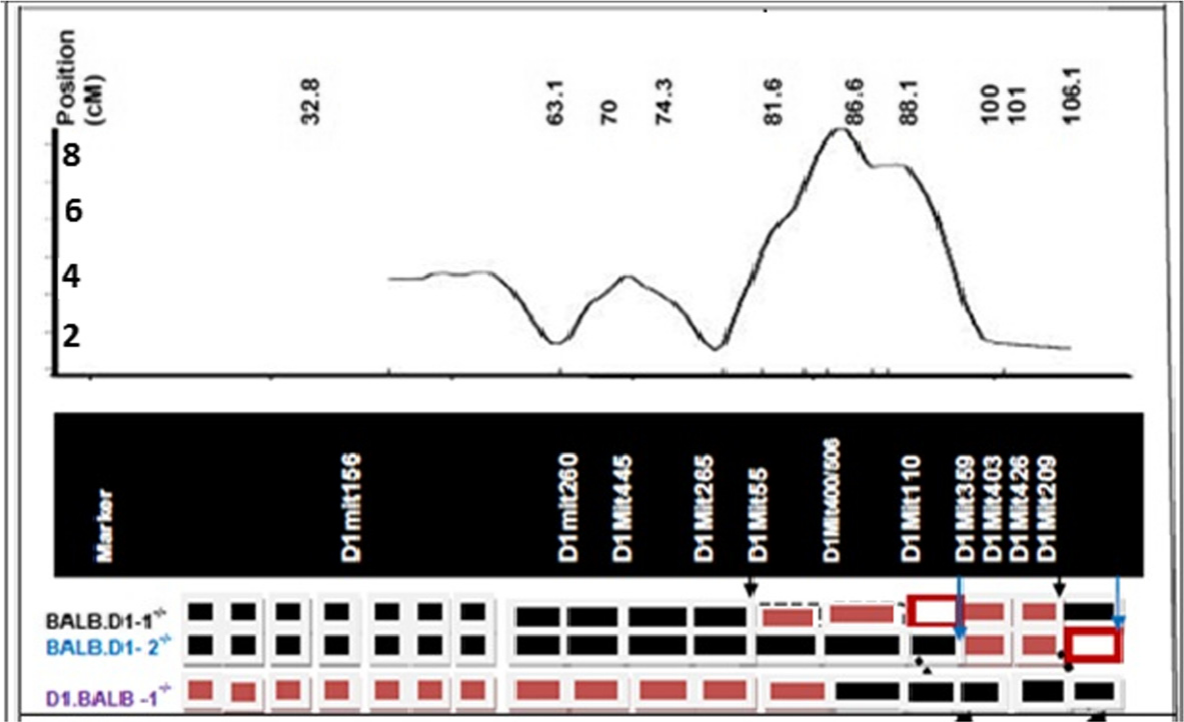
**Genomic region of the QTL on chromosome 1 based three congenic strains.** The top panel represents the location of initial mapping of the QTL for arthritis previously identified using the F2 generation. The numbers of the left of the vertical bar indicates the LOD score. The bottom panel is a diagrammatic representation of the transferred genomic regions (Red boxes) from DBA/1^−/−^ into BALB/c^−/−^ after the generation of 2 congenic strains. The genomic region from DBA/1 in BALB.D1-1^−/−^ is flanked by two markers, D1Mit55 and D1Mit209. The transferred genomic regions (Black boxes) from DBA/1^−/−^ into BALB/c^−/−^ in congenic strain BALB.D1-2^−/−^ is flanked by marker D1Mit359 and the distal end of the chromosome. The two red cycled white boxes indicate where the borders of the QTL are not clearly defined. The genomic region from BALB/c in D1BALB-1^−/−^ is flanked by markers, D1Mit400/506 at the distal end.

At generation of N6, we obtained mice with all DBA/1^−/−^ markers except for those in the region between D1Mit506 and the distal end on chromosome 1 where the markers showed a heterozygous genotype. We back crossed the selected N6 to DBA/1^−/−^ for another generation. The mice then were intercrossed among the N7. The mice with homozygous genotypes of BALB/c^−/−^ in the region between D1Mit506 and the distal end on chromosome 1 then were bred together to form the new congenic strain called D1.BALB-1. The transferred region from BALB/c^−/−^ is from 86.6 cM (D1Mit506) to the distal end of chromosome 1 (Figure 1). The new congenic strains were then observed for the development of spontaneous arthritis.

### Phenotype of Congenic D1.BALB-1 mice

**Figure 2:**
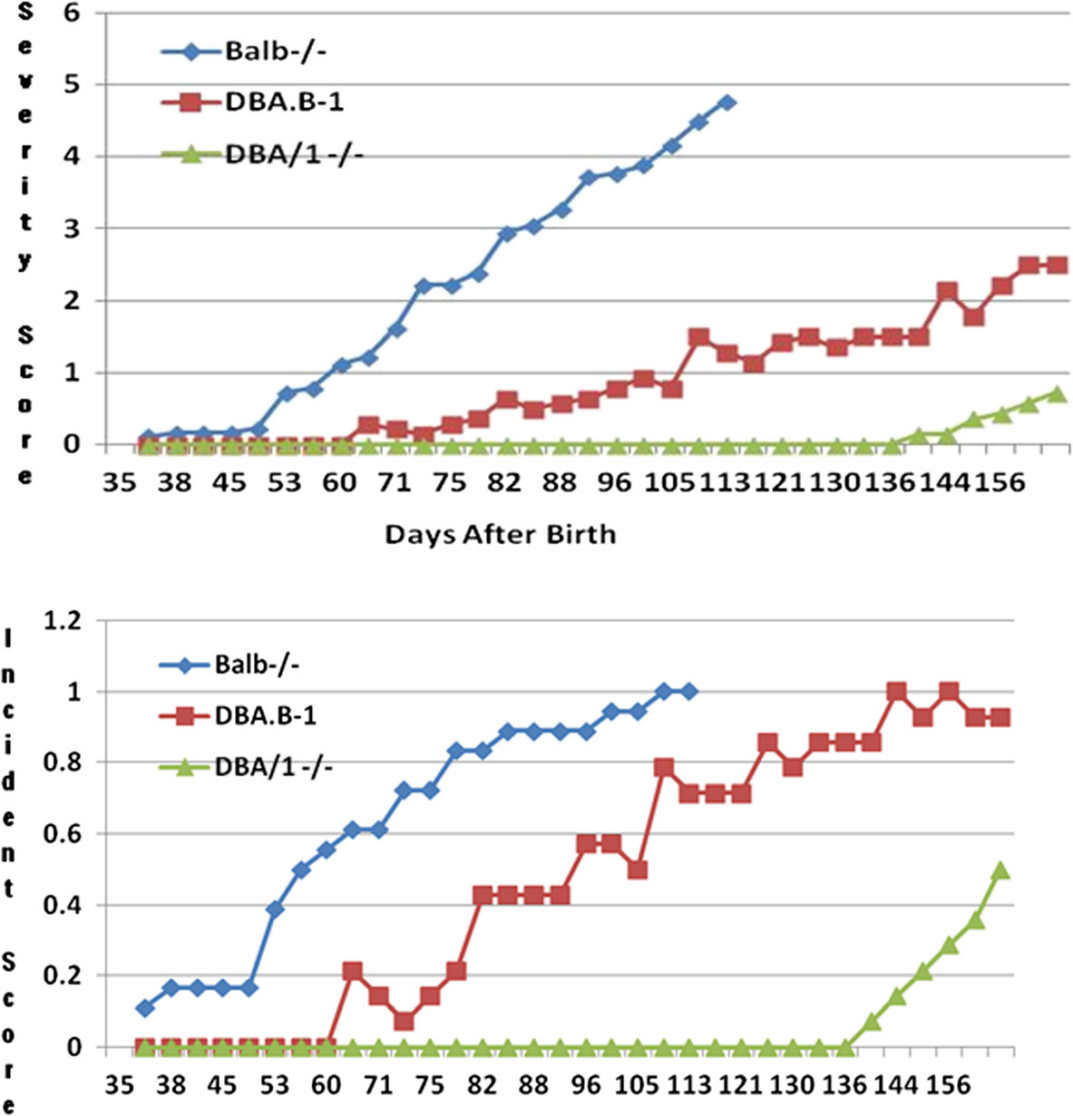
**Arthritis in congenic strains DBA.B-1** ^**−/−**^ **(left Average score (severity), right Incidence) relative to BALB/c** ^**−/−**^ **and DBA/1** ^**−/−**^ **mice.** BALB/c^−/−^ mice reached maximum levels of disease incidence and severity earlier than DBA.B-1^−/−^ mice, which did not reach their maximum levels until week 18. Disease severity measurements are expressed as a percentage of the maximal total score.

None of the DBA/1^−/−^ mice developed arthritis after the age of 4 months. The congenic DBA.B1-1 mice showed mild arthritis on the hind paws as early as 10 weeks, however the joint inflammation was transient. This pre-inflammation period was much longer than that that seen in BALB/c^−/−^ [4,5] in which a clear arthritis phenotype was seen around day 45 (Figure 2). We obtained a stable arthritis phenotype from DBA.B1-1 around 12 weeks of age. Disease expression in 14 mice from each DBA.B1-1 and DBA/1^−/−^ was recorded respectively. Mice were scored twice weekly for disease on each paw, and the scores for each paw were added together to calculate the cumulative clinical score for five months (Figure 2). The arthritis severity in 14 mice reached to 2.5 (Figure 2A), and incidence was almost 100% in five months (Figure 2B). Compared to 14 mice from DBA/1^−/−^, the congenic strain had significantly increased severity and incidence of SAD (P value = 5.33637E-10 and 9.68472E-12, respectively).

### Candidate genes within the QTL region

The position of transferred genomic region from BALB/c^−/−^ to the congenic strain DBA.B1-1 is similar to the previous two congenic strains, in which a genomic region from DBA/1^−/−^ into two congenic strains of BALB/c^−/−^ background [5]. Because of the protection function in both the two previous congenic strains, the susceptibility of the current congenic strain, and the location similarity of transferred genomic fragment in these three congenic strains, we assume that the same gene(s) that regulate the spontaneous arthritis in these strains. By comparing the genetic markers in these three congenic strains, the region of interest can be reduced to a final common transferred region (Figure 1). The estimated size of transferred regions in the DBA.B1-1 is from 163306348 bp (D1mit506) to the distal end. Previously, the two congenic strains of BALB/c^−/−^ background are between 155166854 bp (D1mit55) and 191493284 (D1mit209) in BALB.D1-1 and between 177285202 bp (D1mit 359) and the distal end in BALB.D1-2 (Figure 1). The new data from the DBA.B1-1 confirm our previous estimation of the genomic size of the QTL region: The minimum size of transferred genome region is between 177285202 bp (D1mit 359) and 191493284 (D1mit209). The maximum size is between (167758517 bp (D1mit110) and the distal end.

### Histological confirmation of phenotype of DBA.B-1 pathology

**Figure 3:**
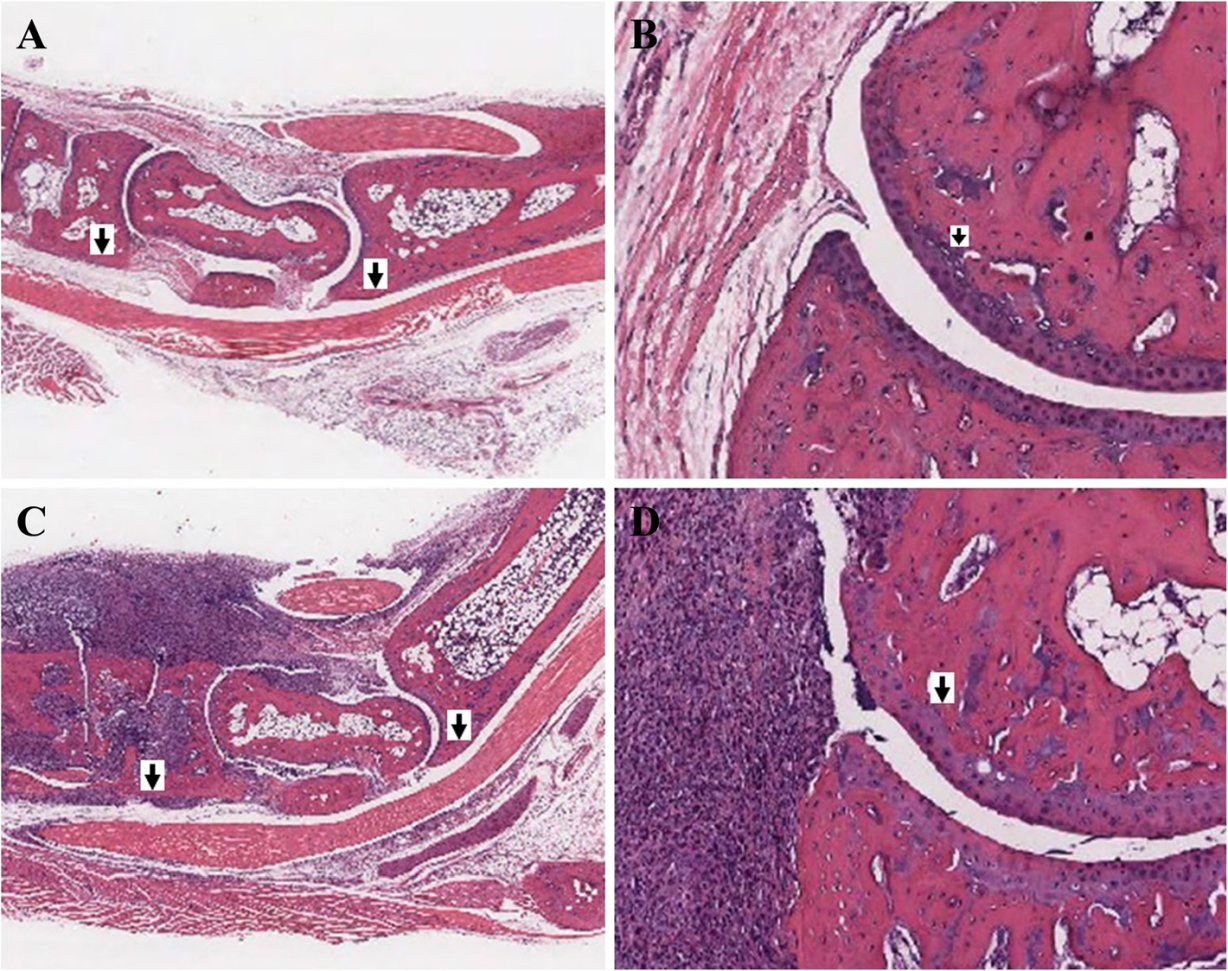
**Comparison of arthritis severity in a DBA/1** ^**−/−**^ **(A, B) and Congenic DBA.B-1** ^**−/−**^ **mice (C, D).** Panels **A** and **C** are cross section of the ankle joint. Panels **B** and **D** are higher power views to illustrate the development of an early erosion in the DBA.B-1^−/−^ mouse whereas the comparable area of the DBA/1^−/−^ mouse shows only synovitis without erosive disease.

**Figure 4:**
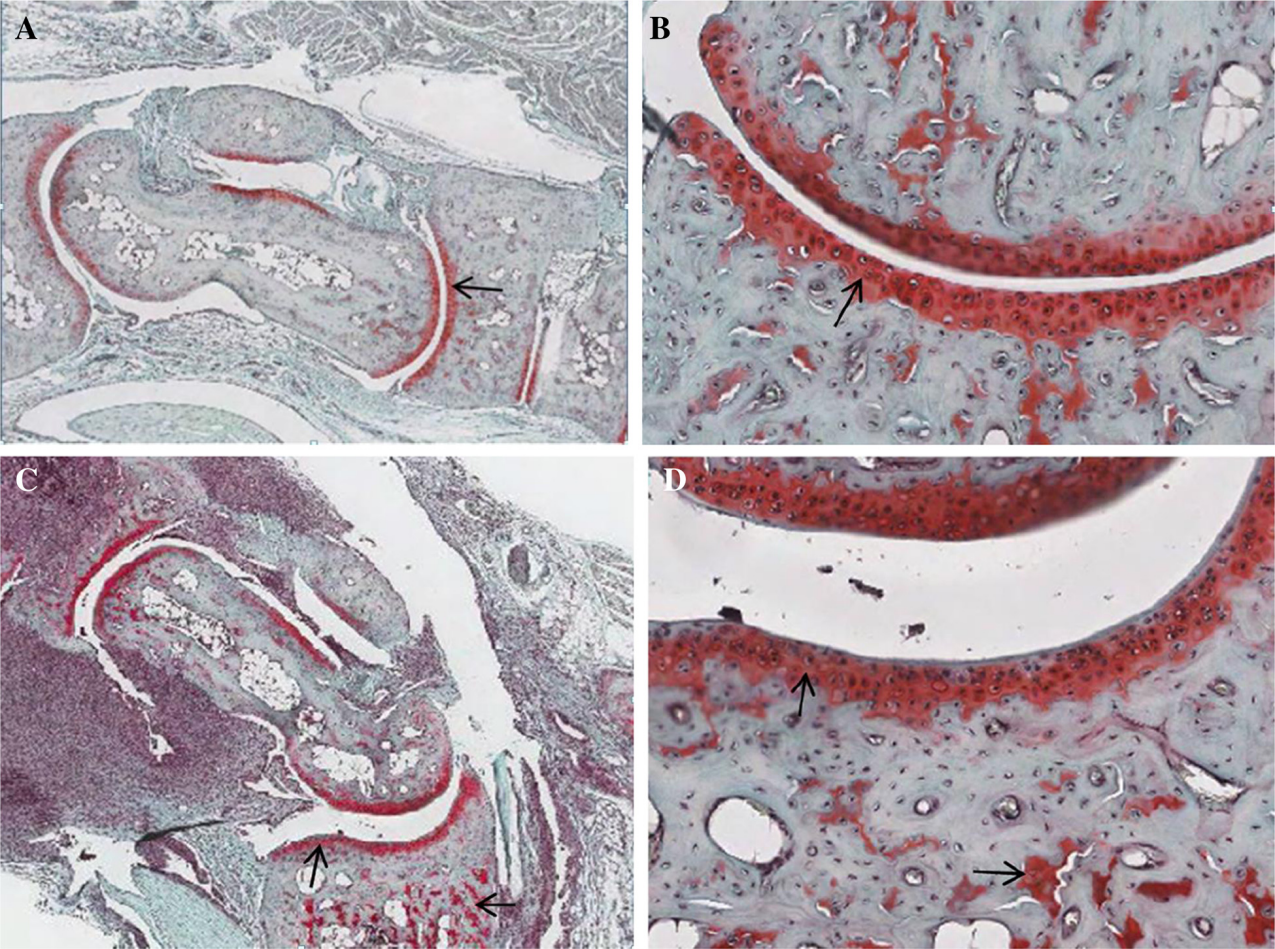
**Comparison of Safranin-O/Fast-Green staining of mouse articular cartilage surface of hind joints, showing the proteoglycan stained by Safranin-O (red) in cartilage matrix in a 16 week old DBA/1** ^**−/−**^ **mouse (A,B) and a congenic DBA.B-1** ^**−/−**^ **mouse (C,D).** Arrow indicates the decreased Safranin-O staining in articular cartilage in congenic DBA.B-1^−/−^ mouse joint **(C,D)**, as compared to that observed in a DBA/1^−/−^ mouse **(A,B)**.

Histological analysis of the ankle joints in congenic DBA.B-1 mice displayed synovial, periarticular inflammation, cartilage matrix destruction and bone erosion (Figure 3). Previously, our congenic BALB/c^−/−^ mice showed pathological erosion at 7 weeks of age [4,5]. Shown in Figure 3 is the histologic examination of a hind limb from a DBA/1^−/−^ compared to a DBA.B-1 ^−/−^ mouse at 16 weeks of age. While the DBA/1^−/−^ appears normal the DBA.B-1 ^−/−^ showed mild erosion in ankle joint. Figure 4 shows the comparison of Safranin-O staining cartilage in 16 weeks old of joints in DBA/1^−/−^ mouse (a,b) and Congenic DBA.B-1^−/−^ mouse(c,d). Data indicates there is decreased Safranin-O staining in articular cartilage in congenic DBA.B-1^−/−^mouse, as compared to that in DBA/1^−/−^ mouse.

**Figure 5:**
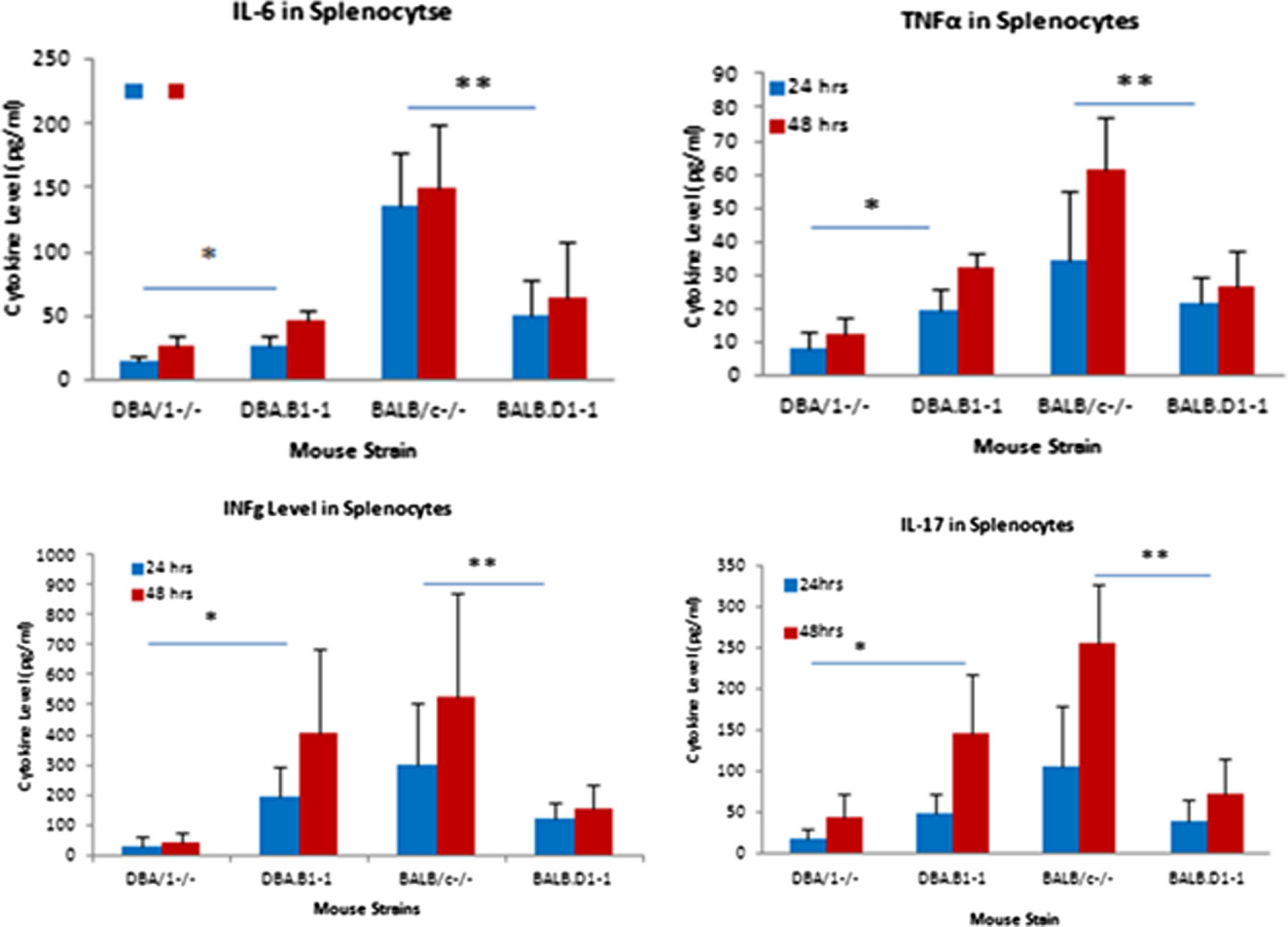
**Production of cytokines by spleen cells from 4 month old mice.** Cells were harvested from individual mice of each strain and cultured with CD3/CD28 stimulation beads. Culture supernatants were harvested after 48 hours and assayed for the cytokines indicated. Each bar represents the mean and standard deviation of 3 biologic replicates. Differences between groups were calculated by Student’s T test.

### Cytokine expressions in congenic strain

To determine if the differences were due to higher generalized T cell responsiveness in the congenic DBA.B-1 strain, we measured the cytokines in congenic mice compared with the DBA/1^−/−^ arthritis resistant and BALB/c^−/−^ susceptible strains. Splenocytes and lymphocytes from each strain were cultured with anti-CD3/CD28 expansion beads for 48 hours, and cytokine levels were measured by Milliplex kit. The levels of cytokines in the respective congenic strains and the two parental strains are shown in Figure 5. These data show that the susceptible parent strain, BALB/c^−/−^, has the highest levels of four cytokines, Il-6, Tnf-α, Ifn-γ and Il-17 while the resistant parent strain, DBA/1^−/−^, has the lowest levels of these cytokines. The levels of these cytokines in congenic strains are in between the two parental strains. Particularly, the expression level of Il-17 in spleen of congenic DBA/1^−/−^ mice is at levels 10-fold greater than that in the DBA/1^−/−^ arthritis resistant strain.

## Discussion

Our data indicated that the QTL fragment on chromosome 1 for susceptibility to spontaneous arthritis works in both directions. Previously, when we transferred a fragment of DNA at QTL locus from the DBA/1^−/−^ strain onto a BALB/c^−/−^ strain background, arthritis was delayed in onset and was less severe [5]. With the new congenic strain, we transferred the similar fragment at the QTL region from BALB/c^−/−^ strain into DBA/1^−/−^ strain background, in contrast to the two other congenic strains, the effect seems to be an increased risk of the disease. Although congenic breeding has been used widely to confirm the function of a QTL and define the genomic region of the QTL, almost every study only transferred the DNA fragment in one direction, thus, transferring a piece of genomic DNA from one parental strain to the other. Our two-way transfer strategy coupled with congenic breeding shows the importance of the source of transferred DNA and the interaction between QTL region and the whole genome in a specific strain. Thus, depending on the genotypes, the genomic DNA from a QTL locus can either reduce or increase susceptibility to the disease.

Our work also indicated the importance of interactions between genotype and genomic background. Animal models have been widely used for the studies of molecular pathways of complex human diseases. However, most animal models are homozygous strains with a unified genomic background. Most studies with single animal models are used as evidence of applicability to humans, yet many of them fail in human trials. Testing the function of a gene or genetic locus of interest in multiple strains under variety genomic backgrounds would allow us to understand their scope and variability in the regulation of disease. In addition, moving a DNA fragment into different genomic backgrounds will enhance our understanding of the molecular mechanisms of the candidate gene(s) within the QTL region.

Our previous analysis indicated that the QTL did not result changes in Il6 and Tnfα or Il-17 based on expression levels in the splenocytes used for analysis [5]. However, when splenocytes were isolated, cultured, stimulated by anti-CD3/CD28 beads and analyzed for cytokine expression by quantitation of protein levels, it was evident that there were different levels of expression based on the genotype. This data suggests that these cytokines may be coordinately regulated by genes within the QTL. In particular, because spontaneous development of arthritis in IL-1Ra-deficient mice has been proven to be Il-17 dependent [6,7], it is likely that the spontaneous arthritis in congenic DBA.B-1^−/−^ strain is specifically related to Il-17 expression. How the QTL interact with these cytokines will need further study.

Previously, our analysis of candidate genes identified 4 candidate genes from the total of 115 differentially expressed genes within the QTL region [5]. Those 4 genes are Fcer1g, Fcgr3, Ifi202b, and Kmo. Interestingly, Fcer1g, Fcgr3 are both show a correlation with gene expression of Il1rn as well as differential expression between spontaneous arthritis mice and healthy BALB/c mice. While Fcer1g and Ifi202b has never been connected to any of Il6, Tnfα, Il-17 and Il-1, Fcgr3 has been linked to Il6 [8] Tnfα [9] in the same gene network. In addition, Kmo has been reported to mediate inhibition of Th-17 differentiation [10]. Therefore, future detailed study for their roles in the spontaneous arthritis in this mouse model may provide important information on their potential roles in the spontaneous arthritis.

The multiple congenic strains for this QTL not only confirm the function of the QTL but also clearly defined the genomic region of the gene(s) that regulates the susceptibility to spontaneous arthritis. With the genomic information of transferred fragment in the new congenic strain DBA.B-1 and the previously two congenic strains under the BALB/c^−/−^ background, the genomic region that contain the candidate gene is firmly defined. One of the major benefits of the congenic strains is to overcome the noise of whole genomic background, which makes difficult to identify the key molecular pathway that regulates the trait of interest. For example, our previous study identified differences of thousands of genes between the susceptible stain BALB/c^−/−^ and DBA/1^−/−^ backgrounds. These congenic strains lay the foundation for the identification of the candidate gene(s) and further understanding of the molecular mechanism of the regulation of spontaneous arthritis using this important mouse model.

## Conclusions

In summary, our study indicates that the effect of the QTL on SAD is clearly detectable in both directions. It clears the way for the measurement of disease phenotype in our positional cloning process. Our two-way transfer of QTL fragments into different genomic backgrounds also is novel. Most important, these congenic strains provide a clue to the potential mechanism of the QTL action and provide valuable resources for the study of molecular mechanism of spontaneous arthritis.

## Methods

### Mice

All mice have been maintained in the Veterinary Medical Unit of the Memphis Veterans Affairs Medical Center. BALB/c^−/−^ spontaneous arthritis mice and DBA/1^−/−^ resistant mice were established in VA animal facilities. Experimental animal procedures and mouse husbandry were performed in accordance with the National Institutes of Health’s Guide for the Care and Use of Laboratory Animals and approved by the VAMC Institutional Animal Care and Use Committee.

### Congenic breeding procedure

The congenic breeding procedure is similar to our previous reported procedure for the congenic strains under BALB/c^−/−^ background [5]. Genomic DNA was extracted from tissues obtained by ear punch. A total of 123 microsatellite markers were selected for genotyping of progeny to assist with identifying the most informative backcrosses for breeding. Briefly, BALB/c^−/−^ were crossed with DBA/1^−/−^ to produce heterozygous (F1) mice; The F1 progeny were backcrossed to DBA/1^−/−^ for 7 generations. In each generation, the individual mice with the fewest genomic markers for BALB/c^−/−^ background but with the QTL region from the BALB/c^−/−^ on chromosome 1 were selected for the next generation. At the end of the sixth generation it was determined that selected mice were homozygous for DBA/1^−/−^ background but contained heterozygous BALB/c^−/−^ markers in the region of the QTL. These mice were then interbred to generate homozygous DBA/1^−/−^ congenic with a BALB/c^−/−^ homozygous region within the QTL. Ultimately we obtained a congenic strain DBA.B1-1^−/−^. Mice were screened periodically for mutation in IL-1rn.

### The phenotype of new congenic strain

Disease phenotype in the new congenic strain was evaluated together with a control strain DBA/1^−/−^ which is a resistant strain (diagram 1). Mice were maintained in standard microisolator cages for 23 weeks. The severity of arthritis was graded for each paw on a scale of 0 to 4 for the degree of redness and swelling [3]: grade 0 = no evidence of swelling; grade 1 = mild swelling of the joint and/or redness of the footpad; grade 2 = obvious joint swelling; grade 3 = severe swelling of entirety and grade 4 = limb burned out and deformed. In the “The phenotype of new congenic strain” of the Methods section, we added the following sentences: “We have to use the DBA/1^−/−^ as the control because in the congenic strain the genomic region of the QTL from BALB/c^−/−^is transferred into the DBA/1 J−/− background. Most importantly, DBA/1^−/−^ is resistant to the spontaneous arthritis while BALB/c^−/−^ is susceptible to the spontaneous arthritis. Wild type BALB/c and DBA/1 mice do not develop spontaneous arthritis [3,5,11].

### Histology

For histological assessment of arthritis, total ankle joints from 16-week-old mice were isolated and fixed in 10% phosphate-buffered formalin; then decalcified in 10% EDTA-4Na and embedded in paraffin. Tissue sections of 5 μm were stained with H&E to examine the inflammatory cell filtration and chondrocyte death. To study proteoglycan (PG) depletion from the cartilage matrix, sections were stained with Safranin O, followed by counterstaining with fast green. Each parameter was scored on a scale from 0 to 3 by 2 independent observers in a blinded manner [12].

### Cytokine measurements

To determine levels of the cytokines Il-6, Tnf-α, Ifn-γ and Il-17 in serum spleen and lymph nodes supernatant samples, Millipore multianalyte technology was used in combination with Milliplex kit (Millipore, MA). Cytokines were measured in 25 ul of sample, supernatant was used directly. The sensitivity of the Milliplex kit was <3 pg/ml [5].

## Abbreviations

IL-1RA: Mice deficient in IL-1 receptor antagonist protein
BALB/c^−/−^: Interleukin-1 receptor antagonist (IL-1rn) -knockout BALB/c mice
DBA/1^−/−^: Interleukin-1 receptor antagonist (IL-1rn) -knockout DBA/1 mice
